# P130Cas Attenuates Epidermal Growth Factor (EGF) Receptor Internalization by Modulating EGF-Triggered Dynamin Phosphorylation

**DOI:** 10.1371/journal.pone.0020125

**Published:** 2011-05-18

**Authors:** Yong Seok Kang, Wook Kim, Yun Hyun Huh, Jeomil Bae, Jin Soo Kim, Woo Keun Song

**Affiliations:** 1 Department of Life Science, Bio Imaging and Cell Dynamics Research Center, Gwangju Institute of Science and Technology, Gwangju, Korea; 2 Diabetes Section, National Institute on Aging, National Institutes of Health, Baltimore, Maryland, United States of America; Kings College London, United Kingdom

## Abstract

**Background:**

Endocytosis controls localization-specific signal transduction via epidermal growth factor receptor (EGFR), as well as downregulation of that receptor. Extracellular matrix (ECM)-integrin coupling induces formation of macromolecular complexes that include EGFR, integrin, Src kinase and p130Cas, resulting in EGFR activation. In addition, cell adhesion to ECM increases EGFR localization at the cell surface and reduces EGFR internalization. The molecular mechanisms involved are not yet well understood.

**Methodology/Principal Findings:**

We investigated the molecular mechanism by which p130Cas affects the endocytic regulation of EGFR. Biochemical quantification revealed that cell adhesion to fibronectin (FN) increases total EGFR levels and its phosphorylation, and that p130Cas is required for this process. Measurements of Texas Red-labeled EGF uptake and cell surface EGFR revealed that p130Cas overexpression reduces EGF-induced EGFR internalization, while p130Cas depletion enhances it. In addition, both FN-mediated cell adhesion and p130Cas overexpression reduce EGF-stimulated dynamin phosphorylation, which is necessary for EGF-induced EGFR internalization. Coimmunoprecipitation and GST pull-down assays confirmed the interaction between p130Cas and dynamin. Moreover, a SH3-domain-deleted form of p130Cas, which shows diminished binding to dynamin, inhibits dynamin phosphorylation and EGF uptake less effectively than wild-type p130Cas.

**Conclusions/Significance:**

Our results show that p130Cas plays an inhibitory role in EGFR internalization via its interaction with dynamin. Given that the EGFR internalization process determines signaling density and specificity in the EGFR pathway, these findings suggest that the interaction between p130Cas and dynamin may regulate EGFR trafficking and signaling in the same manner as other endocytic regulatory proteins related to EGFR endocytosis.

## Introduction

Signaling via the ubiquitously expressed epidermal growth factor receptor (EGFR) is involved in the regulation of cell motility, proliferation, survival and differentiation [1]–[3]. Ligand-dependent asymmetric dimerization of EGFR results in activation of EGFR tyrosine kinase and *trans*-autophosphorylation [4], [5]. The autophosphorylation sites in the activated EGFR then act as nucleation sites for the formation of specific receptor-signaling protein complexes and downstream kinase (e.g., Erk, Akt) cascades that initiate cell context-specific signaling pathways [6], [7].

EGF ligand induces the translocation of activated EGFRs to clathrin-coated pits and their rapid internalization into early endosomes. Endosomal sorting of EGFRs is followed by lysosomal targeting and receptor degradation. EGFR internalization is therefore thought to be a major pathway via which the receptor is downregulated. Alternatively, endosome-associated EGFRs may continue to interact with compartment-specific signaling proteins. Thus the endocytic trafficking of EGFR determines the duration, intensity and specificity of its signal transduction [8]–[13]. In short, endocytosis likely plays multiple roles in the regulation of signaling via EGFRs.

A large number of regulatory proteins are involved in EGFR internalization and signaling [1], [12], [14]. Dynamin GTPase, one of the critical regulators of endocytic EGFR internalization, catalyzes membrane fission and vesicle budding [15]. Dynamin K44A (Lys44→Ala) mutation, which reduces dynamin GTPase activity, inhibits EGFR internalization and, in turn, increases total EGFR levels. In addition, the K44A dynamin mutant reduces specific tyrosine phosphorylation of EGFR and alters downstream signaling, including signaling by Erk [16], [17]. Knocking down key protein components of clathrin-mediated endocytosis, such as clathrin and AP-2, also affects EGFR signaling activities [13]. In addition, hSef binding to EGFR and Vav2, which interact with Gapvd1, modulates EGFR internalization and trafficking, and also affects EGF signaling [10], [18].

Integrin also regulates EGFR internalization and signaling [19]–[21]. Cell adhesion to fibronectin (FN) induces slight increase of EGFR [21] and formation of macromolecular complexes comprised of EGFR, integrin, p130Cas and Src kinase. Upon complex formation, specific EGFR tyrosine residues, including Tyr 845, are phosphorylated, thereby activating EGFR signaling [20], [21]. In contrast to the internalization of activated EGFRs induced by EGF binding, cell adhesion-induced EGFR activation increases localization of EGFRs at the cell surface [20]. The mechanism by which integrin-EGFR crosstalk attenuates EGFR internalization is not well understood, however.

P130Cas is an adaptor protein participating in cell adhesion, motility and transformation [22], [23]. P130Cas has multiple protein-protein interaction domains, including a SH3-domain, a large tyrosine kinase substrate binding domain (SD), and a Src-binding domain (SBD) [22], [24]–[26]. The SDs are characterized by fifteen tyrosine-Xaa-Xaa-proline (YXXP) motifs that are regarded as major sites of adhesion-dependent phosphorylation [24], [25]. The finding that knocking out p130Cas diminishes adhesion-induced EGFR phosphorylation indicates that p130Cas is a functional mediator of integrin-EGFR crosstalk [20]. Moreover, overexpression p130Cas is, by itself, sufficient to induce ligand-independent EGFR phosphorylation of Tyr 845 [27]. However, the mechanism by which p130Cas contributes to the EGFR signaling pathway remains unclear.

The objective of the present study was to investigate the role of p130Cas in the EGFR internalization pathway. Here we show that p130Cas promotes EGFR activation and increases total EGFR levels under conditions of FN-mediated cell adhesion. In addition, p130Cas inhibits EGF-induced EGFR internalization and dynamin phosphorylation. We also demonstrate that the SH3-domain of p130Cas interacts with the proline-rich domain (PRD) of dynamin, and this interaction is essential for p130Cas-mediated inhibition of dynamin phosphorylation and EGFR internalization.

## Results

### P130Cas enhances EGFR activation in response to cell adhesion

**Figure 1:**
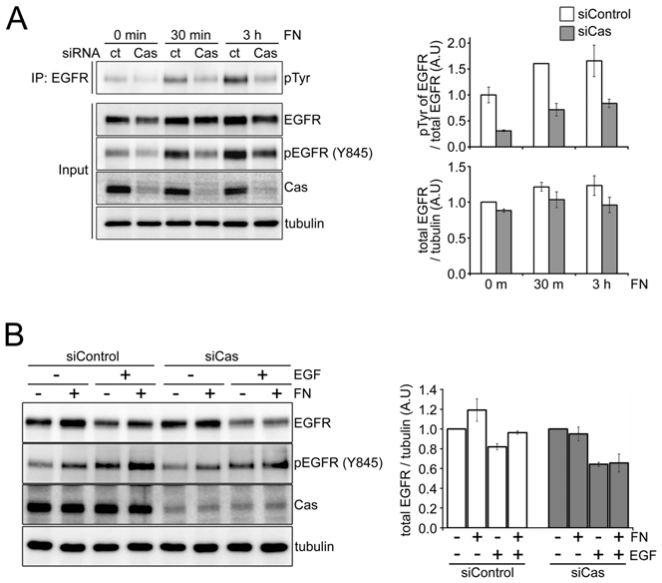
P130Cas enhances EGFR activation and stabilization at the cells surface in response to cell adhesion. (A) A431 cells were transfected with non-targeting (siRNA ct) or p130Cas-specific siRNA (siRNA Cas) duplexes and cultured for 60 h. The cells were then serum starved for 12 h, incubated in suspension (Sus) for 1 h, and plated on FN for 0 min, 30 min or 3 h. Tyrosine phosphorylation of EGFR was analyzed by immunoblotting EGFR immunoprecipitates with an anti-phosphotyrosine antibody (pTyr). Right panel: Graphs showing quantification of EGFR phospho-Tyr levels normalized to total EGFR (top) and total EGFR levels normalized to tubulin (bottom). (B) A431 cells were transfected as described above and then serum starved for 12 h, incubated in suspension (Sus) for 1 h and plated on uncoated or FN-coated dishes for 30 min. The cells were then left untreated or treated with EGF (100 ng/ml) for 2 h. Right panel: Graph showing quantification of total EGFR levels normalized to tubulin.

It is well known that p130Cas is required for cell adhesion-induced EGFR phosphorylation [20], but it was not known whether p130Cas also contributes to cell adhesion-induced increases in EGFR localization at the cell surface. To assess the role of p130Cas in EGFR activation and stabilization at the cell surface induced by cell adhesion, we transfected A431 cells with non-targeting or p130Cas-specific siRNA and examined the effect of knocking down p130Cas. After incubating the transfectants for 60 h, the cells were suspended for 1 h and then plated on FN-coated dishes. Depletion of p130Cas from A431 cells significantly reduced both total EGFR tyrosine phosphorylation (Figure 1A, p-Tyr blot) and EGFR phosphorylation at Tyr 845 (Figure 1A, pEGFR blot). Interestingly, adhesion of control cells to FN elicited a slight increase in total EGFR levels, but adhesion of p130Cas-depleted cells did not.

Given that EGF ligand induces rapid internalization and degradation of EGFRs, we analyzed the effect of p130Cas depletion in the presence of EGF. A431 cells were placed in suspension, and then plated on FN-coated or uncoated culture dishes and treated with 100 ng/ml EGF for 2 h. In control cells, FN-mediated cell adhesion increased total EGFR levels and attenuated EGF-induced EGFR degradation. In p130Cas-depleted cells, by contrast, cell adhesion to FN had no effect on total EGFR levels, and the attenuation of EGFR degradation was abolished (Figure 1B). This suggests that p130Cas plays a regulatory role in EGFR internalization and/or the stable localization of EGFR at the cell surface.

### P130Cas negatively regulates internalization and degradation of EGFR

**Figure 2:**
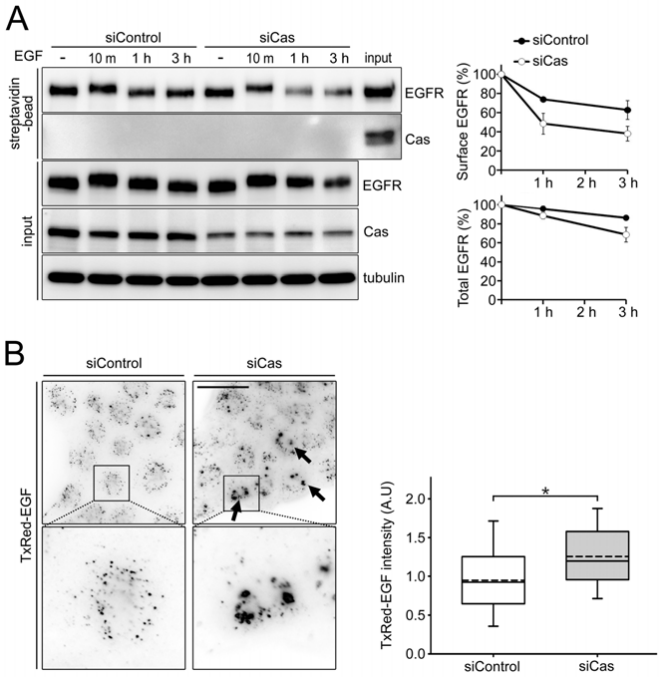
Depletion of p130Cas enhances EGFR internalization and EGF uptake. (A) A431 cells were transfected with non-targeting or p130Cas-specific siRNA, cultured for 60 h and then serum starved for 12 h. After treatment with 100 ng/ml EGF for 10 min, 1 h, or 3 h, cell surface proteins were biotinylated and analyzed as described in the Materials and Methods. Right panel: Graph showing quantification of the remaining surface EGFR (top) or total EGFR (bottom) levels after EGF treatment. Data are from 3 independent experiments. (B) A431 cells transfected with non-targeting or p130Cas-specific siRNA were incubate with Texas Red-EGF on ice for 60 min. After washing with ice-cold DMEM, the cells were incubated at 37°C for 15 min to allow internalization. Samples were then visualized by immunofluorescence microscopy, and the images were captured digitally. Representative images are shown; the arrows indicate the stronger Texas Red-EGF signals seen in p130Cas-depeleted cells. Scale bar, 20 µm. Right panel: Box and whisker plots of Texas Red-EGF (TxRed-EGF) intensity. Fifteen images from each group were selected, and the TxRed-EGF intensity in the cell area was analyzed using MetaMorph software. The data are expressed in arbitrary units (A.U). The middle line in each box indicates the median, the top of each box indicates the 75th percentile, the bottom indicates the 25th percentile, the dotted line indicates average, and the whiskers indicate the extent of the 10th and 90th percentiles, respectively. *P<0.05 (Student's t-test).

To further examine the role of p130Cas in EGFR internalization, we tested the effect of p130Cas on EGF-induced EGFR internalization and subsequent degradation. After biotinylation of cell surface proteins, we traced the effect of EGF on cell surface levels of EGFR. As shown in figure 2A, treating A431 cells with EGF time-dependently reduced levels of EGFR at the cell surface, which is consistent with earlier reports [28]. Interestingly, knocking down p130Cas significantly accelerated the elimination of biotinylated EGFR from the cell surface, implying rapid induction of EGFR internalization. The degradation rate of total EGFR was also increased by p130Cas depletion.

**Figure 3:**
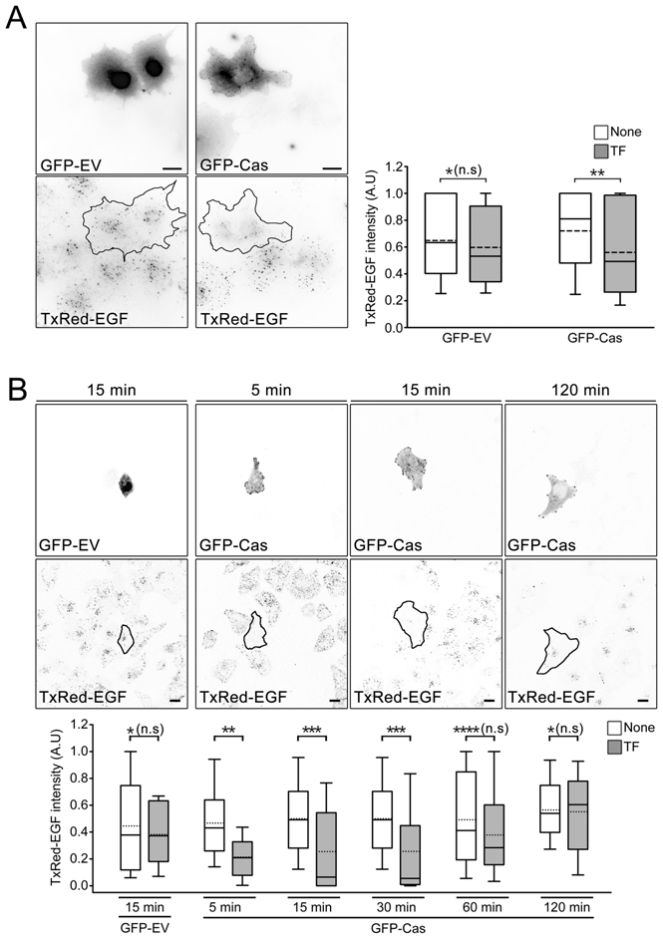
P130Cas overexpression negatively regulates EGF uptake. (A) Left panels: Representative Cos7 cells transfected with empty vector (GFP-EV) or GFP-p130Cas (GFP-Cas) were treated with Texas Red-EGF as described in above. The black lines indicate the transfected cell areas. Scale bars, 20 µm. Right panel: Box and whisker plots of the Texas Red-EGF (TxRed-EGF) intensity per cell. Approximately 40 transfected cells and 50 untransfected neighbour cells were used, and the data are expressed in arbitrary units (A.U). *P>0.3, n.s; not significant, **P<0.05. (B) HeLa cells transfected with empty vector (GFP-EV) or GFP-p130Cas (GFP-Cas) were treated with Texas Red-EGF for the indicated times. Top panels: Representative images; black lines indicate the transfected cell areas. Scale bar, 20 µm. Lower panel: Box and whisker plots of the Texas Red-EGF (TxRed-EGF) intensity per cell. At least 20 transfected cells and >50 untransfected neighbor cells from 15 independent images were analyzed at each time point, and the data are expressed in arbitrary units (A.U). *P>0.5, **P<0.001, ***P<0.01, ****P>0.2.

We next used Texas Red-conjugated EGF to visually track the EGFR. P130Cas-depleted A431 cells exhibited about 35% greater uptake of Texas Red-EGF than control cells (Figure 2B), while overexpression of exogenous p130Cas dramatically reduced EGF uptake. Cos7 cells overexpressing GFP-Cas exhibited less uptake of Texas Red-EGF than their untransfected neighbor cells and cells transfected with empty GFP vector (Figure 3A). We also assessed the effect of overexpressing GFP-p130Cas in HeLa cells coexpressing moderate levels of EGFR. We found that there was less uptake of Texas Red-EGF into HeLa cells overexpressing GFP-p130Cas than into untransfected control cells, but there was no significant difference between transfected or untransfected cells 120 min after EGF treatment (Figure 3B). Taken together, these findings indicate that p130Cas expression negatively regulates EGF-induced EGFR internalization.

### FN-mediated cell adhesion or p130Cas overexpression reduces EGF-dependent dynamin phosphorylation

**Figure 4:**
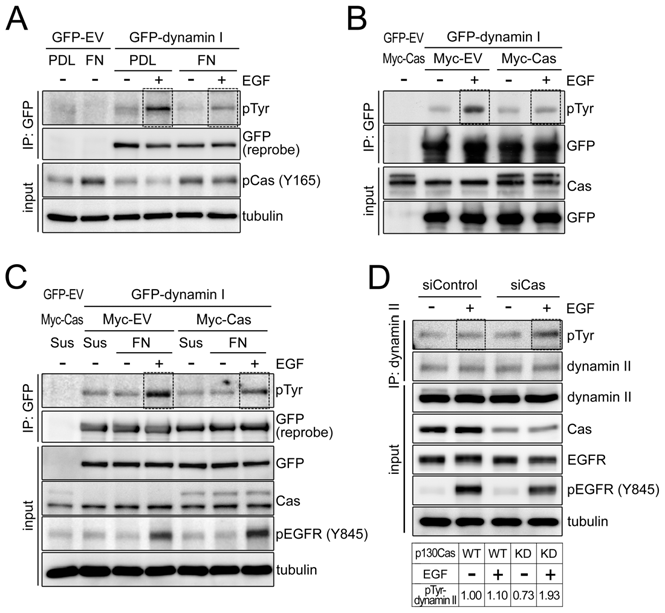
FN-mediated cell adhesion or p130Cas overexpression reduces EGF-dependent dynamin phosphorylation. (A) Cos7 cells were transfected with empty vector (GFP-EV) or GFP-dynamin I and then incubated in suspension (Sus) for 1 h and plated on poly-_D_-lysine (PDL) or fibronectin (FN) for 30 min, with or without 100 ng/ml EGF. GFP-dynamin I immune complexes were generated from the lysate and immunoblotted with antibodies specific to pTyr and GFP (top two blots). In all our experiments, whole cell lysate was also imunoblotted as indicated. A tubulin blot was used as loading control. The dotted box highlights the EGF-induced dynamin phosphorylation. (B) Cos7 cells were transfected with the indicated plasmids and then, after 24 h, were incubated with or without 100 ng/ml EGF. Their lysates were immunoprecipitated with anti-GFP antibody and immunoblotted with anti-pTyr antibody. (C) Cos7 cells were transfected with indicated plasmid set. After 24 h, immunoprecipitation and immunoblotting were carried out as described in (B). (D) A431 cells were transfected with the indicated siRNA and incubated for 60 h. Dynamin II immunocomplexes were precipitated from the lysates and immunoblotted with anti pTyr and anti-dynamin II antibodies. The relative levels of tyrosine phosphorylated dynamin were quantified and normalized with dynamin II level using ImageJ software. The results are depicted in lower panel. WT, wild type; KD, knockdown.

Upon EGF binding to EGFR, dynamin GTPase is activated, which promotes the receptor's internalization [29]. To determine the regulatory mechanism by which p130Cas inhibits EGFR internalization and degradation, we tested whether cell adhesion or p130Cas could alter EGF-induced dynamin phospho-activation. Cos7 cells overexpressing GFP- dynamin I were plated on PDL- or FN-coated dishes, with or without EGF treatment. Subsequent immunoprecipitation revealed that EGF treatment leads to phosphorylation of GFP-dynamin I and that the phosphorylation level is higher in cells plated on PDL than on FN, suggesting cell adhesion can alter EGF-induced dynamin phosphorylation (Figure 4A). To address the possibility that p130Cas affects dynamin phosphorylation, we tested the effect of p130Cas overexpression on EGF-induced dynamin I phosphorylation. As shown in figure 4B, levels of GFP-dynamin I phosphorylation were lower in cells overexpressing Myc-tagged p130Cas (Myc-Cas) than in untransfected cells or cells transfected with empty vector.

Because p130Cas is thought to be important transducer of integrin receptor signaling [22], we tested whether FN adhesion and Myc-Cas overexpression exert a synergistic effect on dynamin I phosphorylation. For this analysis, Cos7 cells were transfected with plasmids, as indicated, and then plated on FN, with or without EGF stimulation. The combination of p130Cas overexpression and FN-mediated adhesion dramatically inhibited dynamin I phosphorylation, suggesting integrin-mediated adhesion and p130Cas may act in concert to regulate dynamin I phosphorylation (Figure 4C). To further confirm the role of p130Cas in the regulation of dynamin phosphorylation, we treated p130Cas-depleted A431 cells with EGF and found that tyrosine phosphorylation of endogenous dynamin II was approximately 2-fold increased by EGF treatment in the p130Cas-depleted cells but 1.1-fold in the control cells (p130Cas wild type) (Figure 4D). These results suggest that p130Cas is able to inhibit EGF-induced phospho-activation of dynamin.

### The p130Cas SH3-domain interacts with the dynamin Pro-rich domain (PRD)

**Figure 5:**
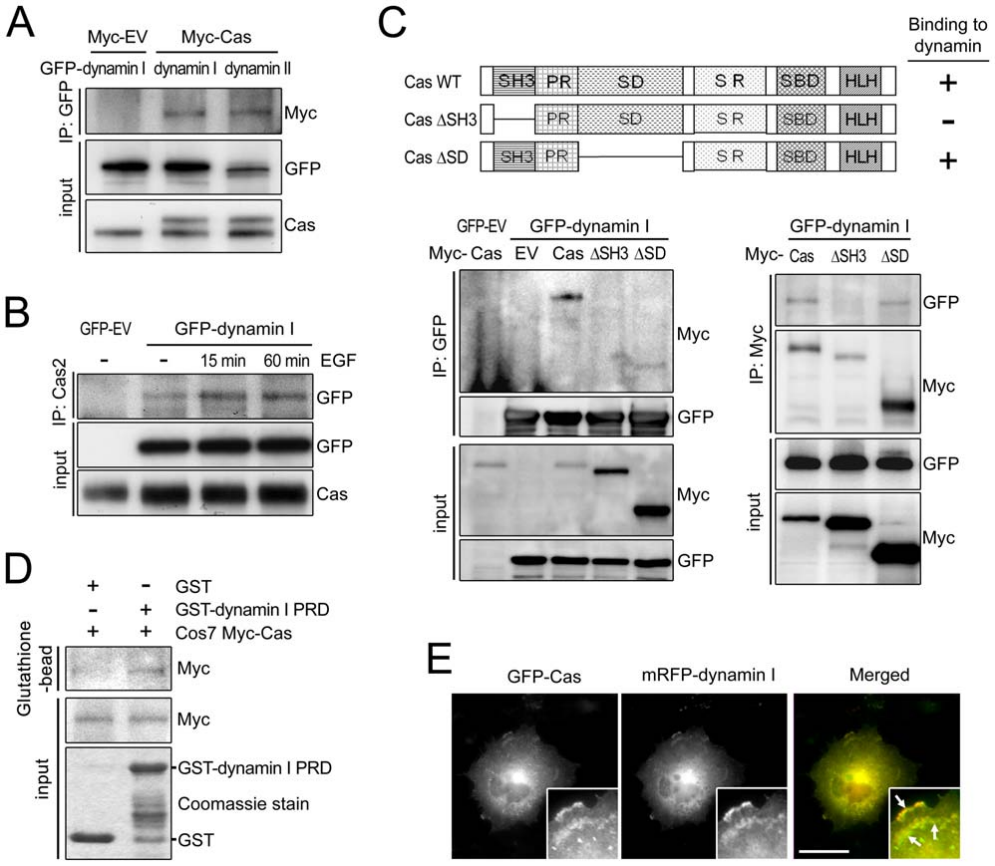
The SH3-domain of p130Cas interacts with the PRD of dynamin. (A) Cos7 cells were transfected with the indicated plasmids, incubated for 24 h, and then lysed for immunoprecipitation with anti-GFP antibody. Immunocomplexes were immunoblotted with anti-Myc antibodies. (B) Cos7 cells were transfected with empty vector (GFP-EV) or GFP-dynamin I and the incubated with or without 100 ng/ml EGF for the indicated times. Cell lysates were immunoprecipitated with anti-Cas antibody (Cas2) and immunoblotted with anti-GFP antibody. Whole cell lysate was also imunoblotted with the indicated antibodies. (C) Schematic representation of p130Cas deletion mutants and the results of interacting domain mapping: WT, wild-type; ΔSH3, deletion of SH3-domain; ΔSD, deletion of deletion of the substrate domain; ‘−’ indicates a lack of detectable interaction; ‘+’ indicates interaction. Lower panels: coimmunoprecipitation of a p130Cas deletion mutant and dynamin I. Cos7 cells were transfected with the indicated constructs, and after 24 h the cell lysates were subjected to coimmunoprecipitation analysis with the indicated antibodies. (D) GST pull-down assay verifying the interaction between p130Cas and dynamin. Myc-Cas was pulled down from the lysates of Cos7 transfectants by a GST-dynamin PRD fusion protein immobilized on glutathione beads. The bound proteins were eluted, subjected to SDS-PAGE and immunoblotted using anti-Myc antibody. (E) Cos7 cells were transfected with GFP-p130Cas (GFP-Cas) and mRFP-dynamin I. After 24 h, the cells were prepared for immunofluorescence analysis as described in the Materials and Methods. The boxed areas are enlarged in the insets.

The existence of p130Cas/dynamin complexes in mammalian tissues was previously demonstrated through coimmunoprecipitation of lysates from the seminiferous tubules and germ cells of adult rats [30]. Bearing that in mind, inhibition of the EGF-induced dynamin phosphorylation by p130Cas prompted us to investigate whether p130Cas interacts with dynamin. Cos7 cells were transiently transfected with GFP-dynamin, with or without a plasmid encoding Myc-Cas. After coimmunoprecipitation, the immunocomplexes were probed for GFP. The result showed that GFP-dynamin I associates with Myc-Cas (Figure 5A) and with endogenous p130Cas (Figure 5B). We then performed coimmunoprecipitation analyses using p130Cas deletion mutants lacking the SH3-domain (Cas ΔSH3) or SD (Cas ΔSD) to identify the p130Cas domain that interacts with dynamin. We found that dynamin primarily bound to the SH3-domain of p130Cas via its PRD (Figure 5C and D). To confirm the direct interaction between p130Cas and dynamin, GST pull-down assays were performed using purified GST-dynamin I PRD and cell lysates from Cos7 cells overexpressing Myc-Cas. As shown in figure 5D, GST-dynamin I PRD was pulled down with Myc-Cas, confirming a specific interaction between p130Cas and the dynamin I PRD.

To further confirm p130Cas-dynamin interaction, we tested whether p130Cas colocalizes with dynamin. After EGF treatment, transiently expressed GFP-Cas and mRFP-dynamin I were readily colocalized near cell edge (Figure 5E, arrows). Similar pattern of co-localization was previously demonstrated by cortactin, a well-characterized binding partner of dynamin [31]. It thus appears that p130Cas can associate with dynamin, and that the p130Cas SH3-domain mediates binding to the dynamin PRD.

### Deletion of the p130Cas SH3-domain restores EGF-induced dynamin phosphorylation and EGF internalization

**Figure 6:**
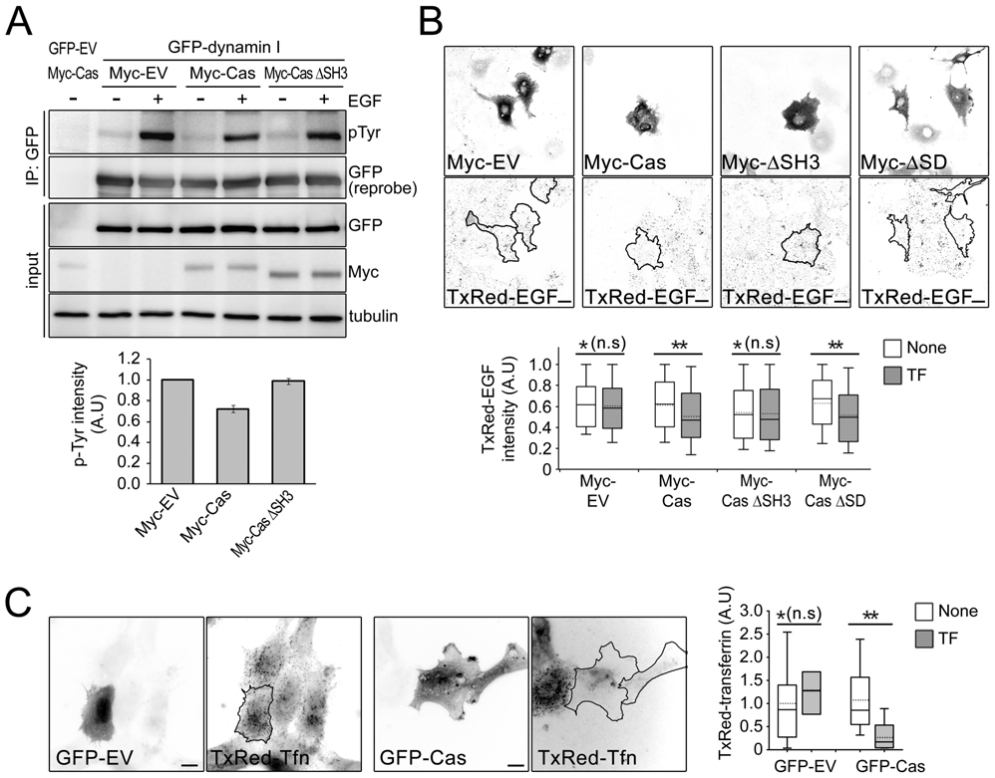
Deletion of the p130Cas-SH3 domain restores EGF-induced dynamin phosphorylation and EGF internalization. (A) Cos7 cells transfected with the indicated plasmids were incubated with or without 100 ng/ml EGF, after which their lysates were immunoprecipitated with anti-GFP antibody and immunoblotted with anti-pTyr and anti-GFP antibodies. Whole cell lysate was also imunoblotted with the indicated antibodies. ΔSH3, deletion of SH3-domain, Lower panel: Graph showing levels of tyrosine phosphorylation of GFP-dynamin I normalized to tubulin. (B) Cos7 cells were transfected with empty vector (Myc-EV), wild-type Myc-p130Cas (Myc-Cas), Myc-p130Cas SH3-domain deletion mutant (Myc-Cas ΔSH3) or Myc-p130Cas SD deletion mutant (Myc-Cas ΔSD) and then treated with Texas Red-EGF as described as above. Upper panels: Representative images; black lines indicates the transfected cell areas. Scale bars, 20 µm. Lower panel: Box and whisker plots of Texas Red-EGF (TxRed-EGF) intensity per cell. For each condition, 37–120 transfected cells and 90–146 untransfected neighbor cells were assessed, and the data are expressed in arbitrary units (A.U). *P>0.2, **P<0.001. (C) HeLa cells transfected with empty vector (GFP-EV) or GFP-p130Cas (GFP-Cas) were treated with Texas Red-transferrin (TxRed-Tfn) as described above. Left panels: Representative images; black lines indicate the transfected cell areas. Scale bars, 10 µm. Right panel: Box and whisker plots of Texas Red- transferrin intensity per cell. Approximately 20 transfected cells and 40 untransfected neighbor cells were used, and the data are expressed in arbitrary units (A.U). *P>0.2, **P<0.005.

Given that p130Cas negatively regulates EGF-induced dynamin phosphorylation and EGFR internalization (Figures 2, 3, 4), we set out to determine the function of the p130Cas-dynamin interaction during dynamin phosphorylation and EGFR internalization. Cos7 cells were cotransfected with GFP-dynamin I and wild-type p130Cas or Cas ΔSH3, after which GFP-dynamin I was immunoprecipitated in the presence or absence of EGF, and the precipitate was immunoblotted with anti-phospho-tyrosine antibody (pTyr). Introduction of wild-type p130Cas into cells reduced EGF-induced dynamin phosphorylation, and overexpression of Cas ΔSH3 restored EGF-induced dynamin phosphorylation to control levels (Figure 6A). Because dynamin phosphorylation directly affected EGFR internalization, we investigated the effect of Cas ΔSH3 on EGF uptake and found that EGF uptake was significantly inhibited by overexpression of p130Cas (Figures 2 and 3). As expected, the inhibition was abolished in the cells expressing Cas ΔSH3, which is unable to interact with the dynamin PRD (Figure 6B). This suggests that the p130Cas-dynamin interaction is required for the inhibitory effect of p130Cas on EGF-triggered dynamin activation and subsequent EGFR internalization.

## Discussion

P130Cas is a well-characterized adaptor/scaffold protein that plays a key role in mediating integrin signaling and regulating extracellular matrix (ECM)-dependent cell migration and cell transformation [22]–[24]. There have been several reports that p130Cas is important for formation of integrin-EGFR complexes, and for modulation of EGF signaling in response to ECM stimuli [20], [22]. In the present study, we demonstrated that p130Cas is also required for FN-dependent EGFR phosphorylation, without stimulation by EGF, and for increases in total EGFR levels stimulated by FN-mediated cell adhesion. In addition, p130Cas tends to stabilize EGFR at the cell surface, even in the presence of EGF. Overexpression of p130Cas in Cos7 and HeLa cells delayed EGFR internalization, as indicated by EGF uptake assays. Conversely, knocking down p130Cas using siRNA in A431 cells enhanced EGFR internalization. To identify the mechanism by which p130Cas negatively regulates EGFR internalization and degradation, we assessed the relation between the activities of p130Cas and dynamin GTPase. We found that p130Cas acting in association with dynamin is able to downregulate EGF-induced dynamin phosphorylation and, in turn, reduce dynamin activity. The novel role of p130Cas in regulating EGFR endocytosis and dynamin activity provides new mechanistic insight into cell adhesion-dependent regulation of EGF signaling.

It was previously shown that ligand activated EGFRs are rapidly internalized and, through endosomal trafficking, are targeted to lysosomal degradation [2], [11], [32]. By contrast, EGFR activation through integrin-mediated cell adhesion tends to reduce EGFR internalization and to increase EGFR levels at the cell surface. Moreover, it was previously reported that p130Cas plays an essential role in cell adhesion-induced EGFR activation [20]. We therefore hypothesized that, like FN-mediated adhesion, p130Cas may contribute to EGFR stabilization at the cell surface. Consistent with that notion, knocking down p130Cas attenuated cell adhesion-mediated EGFR activation as well as adhesion-induced increases in total EGFR levels without influencing EGFR expression (Figure 1B and data not shown). Furthermore, knockdown of p130Cas enhanced EGF-induced EGFR internalization and degradation, while overexpression of p130Cas inhibited EGF uptake, indicating that EGFR internalization is likely regulated by p130Cas (Figure 2 and 3). Given that Neu (also known as ErbB2, Her2) associates with the cell surface EGFR at an early time point after EGF treatment [8], it is interesting to note that depletion of p130Cas in A431 cells dramatically reduced EGFR/Neu dimerization by EGF (Figure S1A).

Previous studies established that proteins regulating EGFR endocytosis can also affect downstream signaling by EGF [14]. For example, hSef overexpression attenuates EGFR degradation and promotes activity in the EGF-triggered Erk pathway [10]. Exogenous Vav2 expression also delays EGFR internalization and stabilizes EGFR at the cell surface, leading to enhanced phosphorylation of EGFR, Erk and Akt [18]. In accordance with previous reports, overexpression of p130Cas in Cos7 cells clearly led to phosphorylation of EGFR and Akt (Figure S1B and C), indicating that p130Cas is able to modulate downstream signaling of EGF as well. However, phosphorylation of Erk, a downstream mediator of EGFR signaling, was unaffected by changes in the p130Cas levels (overexpression or knockdown). This likely reflects the complexity and/or the diversity of the Erk activation pathway in different cell types and signaling contexts. [19], [33].

EGFR internalization is regulated by several mechanisms, including specific ligand binding, EGFR ubiquitination, the binding of the endocytic machinery to EGFR and the participation of other accessory proteins [11], [12], [28], [32], [34]. Src is well established as an endocytosis regulator required for efficient receptor internalization [35]. EGF treatment increases Src-dependent phosphorylation of dynamin, which leads to EGFR internalization and degradation [29]. On the other hand, it is noteworthy that both FN-mediated cell adhesion and the Src-binding domain of p130Cas are able to transiently activate Src kinase [36]–[38], nevertheless ectopic expression of p130Cas together with FN-mediated cell adhesion reduced EGF-induced phosphorylation of dynamin (Figure 4). Thus the mechanism by which integrin-EGFR crosstalk and p130Cas attenuates EGFR internalization may involve differential regulation of Src kinase activity.

Given that the dynamin PRD is a potential target for numerous proteins containing an SH3-domain [15], it seems plausible that p130Cas reduces dynamin phosphorylation by binding to dynamin, thereby interrupting the dynamin-Src association needed for the reaction. This idea is supported by the observation that a p130Cas mutant lacking the SH3-domain (Cas ΔSH3) could not interact with dynamin, nor could it inhibit EGF uptake (Figure 5 and 6). However, it remains to be determined whether p130Cas-mediated inhibition is achieved through interference with the Src-dynamin interaction, or whether p130Cas activity leads to a reduction in dynamin GTPase activity and self-assembly.

Src-dependent phospho-activation of dynamin is a crucial step for several receptor proteins such as β2-adrenergic receptor, transferrin receptor as well as EGFR. [29], [39], [40]. It is therefore possible that p130Cas affects the internalization of other receptors in addition to EGFR. Indeed, we observed that depletion of p130Cas from HeLa cells increased Texas Red-transferrin uptake (Figure 6C). It would be interesting to know whether p130Cas is a common regulator of internalization of a wide spectrum of receptors through its inhibition of receptor-mediated elevation in dynamin activity.

In summary, this work outlines the inhibitory role of p130Cas in EGFR internalization and EGF-stimulated dynamin activation, and provides evidence that p130Cas stabilizes EGFR at the cell surface, thereby regulating its downstream signaling.

## Materials and Methods

### Ethics Statement

All animal experiments were approved by the Gwangju Institute of Science and Technology Animal Care and Use Committee (the permit number: GIST-2008-35).

### Materials

Rabbit anti-phospho-EGFR (Tyr 845), rabbit anti-phospho-Akt (Ser473), mouse anti-phospho-Erk, rabbit anti-EGFR, goat anti-dynamin II and rabbit anti-Neu antibodies were from Santa Cruz Biotechnology (Santa Cruz, CA, USA). Rabbit anti-phospho-p130Cas (Tyr 165) was from Cell Signaling Technology (Beverly, MA, USA). Mouse monoclonal anti-p130Cas (Clone 21) was from BD Biosciences (San Jose, CA, USA). Mouse monoclonal anti-phospho-Tyr (clone 4G10) and mouse monoclonal anti-dynamin I (Hudy 1) were from Upstate Biotechnology (Lake Placid, NY, USA). Mouse monoclonal anti-tubulin was from Sigma (St Louis, MO, USA). Rabbit anti-GFP antibody was generated by immunizing New Zealand White rabbits with the GST-GFP protein and then purified. Mouse monoclonal anti-Myc (clone 9E10) antibody was generated from mouse ascites after hybridoma injection. Rabbit anti-p130Cas (Cas2) was a gift from Dr. Hismaru Hirai (University of Tokyo) [41]. Texas Red-conjugated EGF was from Molecular Probes (Eugene, OR, USA), and Texas Red-conjugated transferrin and recombinant EGF were from Invitrogen (Carlsbad, CA, USA). Human FN and synthetic poly-_D_-lysine (PDL) were from Sigma (St Louis, MO, USA).

### Cell culture and transfections

Cos7 and HeLa cells were obtained from the American Type Culture Collection (ATCC, Manassas, USA), and A431 cells from the Korean Cell Line Bank (KCLB, Seoul, Korea). Cells were maintained in Dulbecco's modified Eagle's medium (DMEM) supplemented with 10% fetal bovine serum (FBS) (Hyclone). Cell culture plates were coated with 20 µg/ml FN or 75 µg/ml PDL as needed. Cover slips were coated with 0.2% gelatin before cell plating.

pGEX4T1 dynamin I PRD, pEGFP dynamin I and pEGFP dynamin II constructs were generously provided by Dr. Sunghoe Chang (Seoul National University College of Medicine). mRFP-dynamin I was a gift from Dr. Pietro DeCamilli (Yale University). pEGFP p130Cas [42], Myc-tagged pcDNA3 wild-type and mutant p130Cas plasmids were described earlier [43]. All transfections were carried out using Lipofectamine (Invitrogen, Carlsbad, CA, USA) or GeneExpresso 8000 (InnoVita, Gaithersburg, MD, USA) according to the manufacturer's instructions.

Cells were depleted of p130Cas using siRNA corresponding to nucleotides 2366–2384 (CCCACAAGCUGGUGUUACU dTdT) of p130Cas [44] (Bioneer, Daejeon, Korea). Transfection of control (nonsilencing fluorescein-labeled siRNA duplex; Bioneer) or anti-p130Cas siRNA was performed in Opti-MEM I medium (Invitrogen) using Lipofectamine 2000 reagent (Invitrogen, Carlsbad, CA, USA), following the manufacturer's instructions. The transfection efficiency for the siRNAs, as determined from images of fluorescein-labeled siRNA duplex, was consistently >95%.

For cell adhesion assays, Cos7 and HeLa cells were serum starved for 12 h, and A431 cells were serum starved 24 h, after which they were detached and replated on culture dishes coated with FN or PDL. The cells were then incubated with or without 100 ng/ml EGF in DMEM for the indicated times. EGF-induced dynamin phosphorylation was performed as described previously [29]. Briefly, cells transiently expressing GFP-dynamin I were serum-starved for 12 h and then pretreated with 100 µM Na_3_VO_4_ for 1 h followed by treatment with 100 ng/ml EGF for an additional 30 min. For Texas Red-EGF and Texas Red-transferrin uptake assays, the cells were serum starved as above, after which they were chilled, washed with cold PBS and incubated with 1 µg/ml Texas Red EGF or 25 µg/ml Texas Red-transferrin for 1 h at 4°C. After washing away the unbound ligand, the cells were rapidly warmed to 37°C for 15 min before being fixed for immunofluorescence analysis.

### Immunoprecipitation and Immunoblotting

Twenty-four hours after plasmid transfection or 60 h after siRNA transfection, the cells were manipulated as described above and then lysed in modified radioimmunoprecipitation assay buffer (50 mM Tris-HCl, pH 7.4, 150 mM NaCl, 1% NP-40, 0.25% sodium deoxycholate, 10 mM NaF, 1 mM PMSF, 1 mM Na_3_VO_4_, 10 µM leupeptin, 1.5 µM pepstatin, and 10 µg/ml aprotinin). The lysates were cleared by centrifugation at 12,000 r.p.m for 10 min at 4°C, after which the appropriate antibody was added to the supernatant and incubated for 4 h or overnight at 4°C. The resultant immune complexes were precipitated with protein A or G-Sepharose (GE Healthcare, Piscataway, NJ, USA) for 3 h. The beads were then washed four times with lysis buffer, suspended in SDS sample buffer, boiled for 10 min, resolved by SDS-PAGE and analyzed by immunoblotting as described previously [45]. Tyrosine phosphorylation of EGFR was analyzed by immunoprecipitation with anti-EGFR antibody, followed by immunoblotting with anti-phospho-Tyr antibody. Phosphorylation of GFP-anti-dynamin I and endogenous dynamin II was detected by immunoprecipitation with anti-GFP or dynamin II antibody, followed by immunoblotting with anti-phospho-Tyr antibody. For analysis of the interaction of GFP-dynamin with wild-type or mutant Myc-p130Cas, the lysates were immunoprecipitated with anti-GFP followed by immunoblotting with anti-Myc antibody. Interaction of GFP-dynamin with endogenous p130Cas was analyzed by immunoprecipitation with anti-p130Cas antibody followed by immunoblotting with anti-GFP antibody. The resultant band intensities were measured using ImageJ software (Ver. 1.43u, NIH, Bethesda, USA).

### EGFR internalization assay

Cells transfected with siRNA or plasmid DNA were serum starved for 12 h and treated with EGF-containing DMEM, as indicated. They were then washed three times with cold PBS and incubated with 0.5 mg/ml Sulfo-NHS-SS-Biotin (Thermo Scientific, Waltham, MA, USA) in a borate buffer (10 mM boric acid, 150 mM NaCl, pH 8.0) for 1 h at 4°C with gentle shaking. The reaction was quenched by adding ice-cold PBS containing 15 mM glycine, after which the cells were washed with cold PBS, lysed and precipitated using streptavidin-agarose resin (Thermo Scientific, Waltham, MA, USA). The precipitates were then immunoblotted with anti-EGFR antibody to detect cell surface EGFR. P130Cas blots served as a negative control of membrane protein biotinylation. Aliquots of cell lysate were taken before the incubation with streptavidin-agarose and analyzed by immunoblotting using the indicated antibodies.

### Immunofluorescence

To detect Texas Red-EGF, GFP-p130Cas, mRFP-dynamin or Myc-p130Cas, the cells were treated as described in the figure legends, fixed, permeabilized, blocked, incubated with the appropriate primary antibody, and stained with Alexa Fluor-conjugated secondary antibody (Invitrogen, Carlsbad, CA, USA). Immunohistochemical detection of Myc-p130Cas protein was carried out using anti-Myc (clone 9E10) as the primary antibody, and nuclei were labeled with Hoechst dye 33258 (Sigma, St Louis, MO, USA). Images were obtained using an Olympus confocal microscope FV1000 (Olympus, Tokyo, Japan) using FV10-MSASW software or a Leica DMR upright fluorescence microscope (Leica Microsystems, Wetzlar, Germany) driven by MetaMorph imaging software (Ver. 6.3r6, Molecular Devices, Sunnyvale, CA). Fluorescence intensities within cells were measured using MetaMorph Offline image analysis software (Ver. 7.6.0.0, Molecular Devices, Sunnyvale, CA).

### GST pull-down assay

GST or GST-dynamin I PRD was expressed in *Escherichia coli* and purified through adsorption onto glutathione-Sepharose 4B beads (GE Healthcare, Piscataway, NJ, USA). Cell lysates were prepared from Cos7 cells expressing Myc-Cas or Myc-only empty vector using the modified radioimmunoprecipitation assay buffer described above. Aliquots (2 mg) of cell lysate were incubated with GST or GST-dynamin I PRD in binding buffer (50 mM HEPES, pH 7.5, 150 mM NaCl, 0.1% Tween 20, 1 mM EDTA, 2.5 mM EGTA, 10% glycerol, 1 mM dithiothreitol, 0.1 mM Na_3_VO_4_, 1 mM PMSF, and protease inhibitors), after which the beads were extensively washed in the same buffer, and the adsorbed proteins were separated by SDS-polyacrylamide gel electrophoresis and visualized by immunoblotting with anti-Myc antibody.

## Supporting Information

Figure S1 **P130Cas modulates EGFR signaling differentially depending on the cell type.** (A) P130Cas depletion reduces Neu-EGFR association in A431 cells. Depletion of p130Cas from A431 cells alters EGF-induced EGFR signaling (e.g., Neu-EGFR dimerization) [8], [46]. A431 cells transfected with non-targeting or p130Cas-specific siRNA were serum starved or stimulated with EGF (100 ng/ml) for 60 min at 4°C and then warmed for 10 min at 37°C. Cell lysates were used for immunoprecipitation or immunoblot analysis with the indicated antibodies. (B) FN-mediated cell adhesion increases phosphorylation of EGFR, Akt and Erk. Cos7 cells were incubated in suspension (Sus) for 1 h and plated on FN for 30 min or 3 h. They were then lysed, subjected to SDS-PAGE and immunoblotted using the indicated antibodies. In all of our experiments, whole cell lysate was also imunoblotted as indicated, and tubulin was used as a loading control. (C) Overexpression of p130Cas enhances FN-induced phosphorylation of EGFR and Akt, but not Erk. Cos7 cells were transfected with empty vector (GFP-EV) or GFP-p130Cas (GFP-Cas) and treated as described in (B).
(TIF)
