# Supplementary figures and images for: P130Cas Attenuates Epidermal Growth Factor (EGF) Receptor Internalization by Modulating EGF-Triggered Dynamin Phosphorylation
Source: PLoS One. 2011 May 18;6(5):e20125. doi: 10.1371/journal.pone.0020125 (PMC3097230; doi:10.1371/journal.pone.0020125)

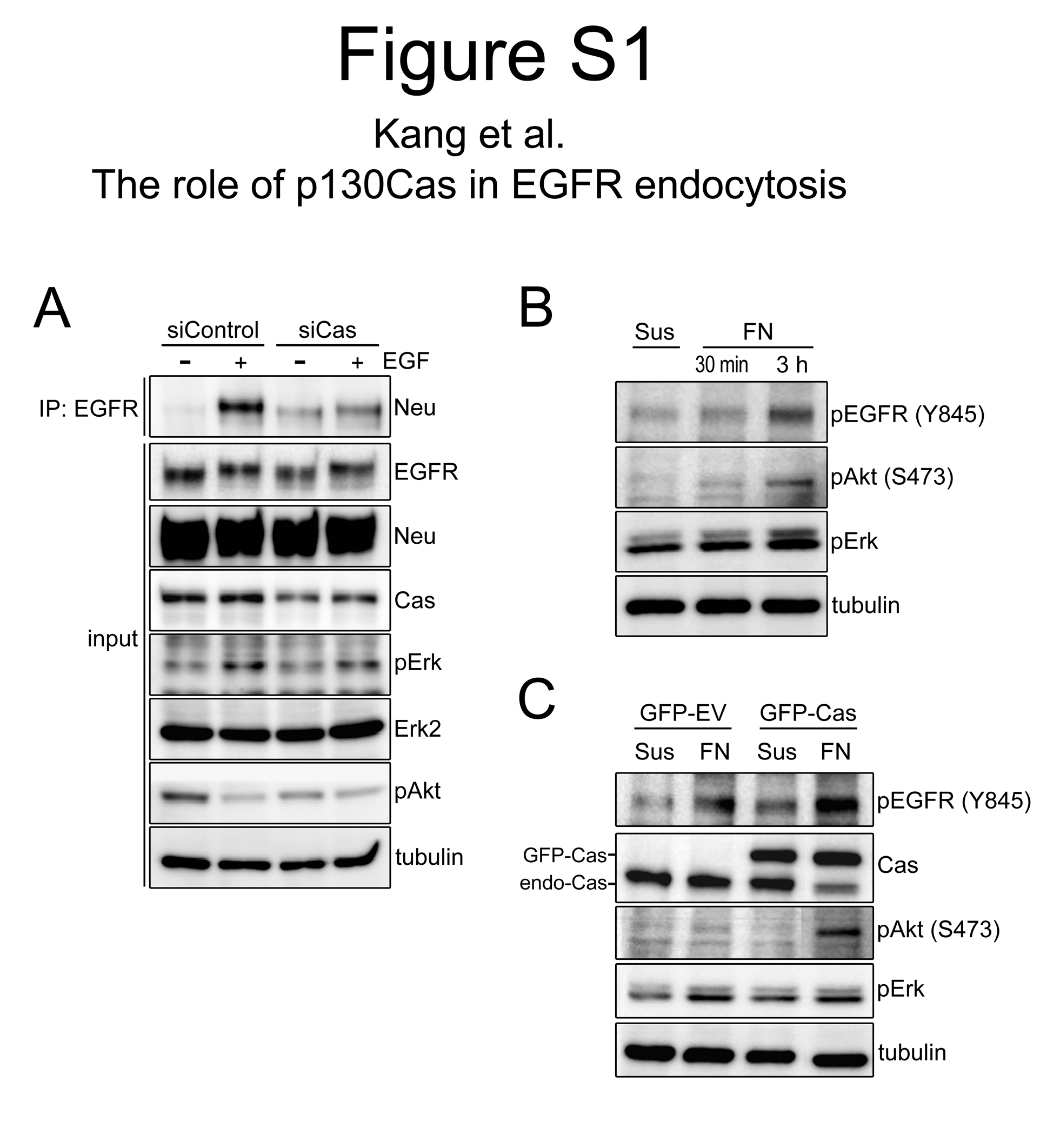

Supplement: Figure S1 — P130Cas modulates EGFR signaling differentially depending on the cell type. (A) P130Cas depletion reduces Neu-EGFR association in A431 cells. Depletion of p130Cas from A431 cells alters EGF-induced EGFR signaling (e.g., Neu-EGFR dimerization) [8], [46]. A431 cells transfected with non-targeting or p130Cas-specific siRNA were serum starved or stimulated with EGF (100 ng/ml) for 60 min at 4°C and then warmed for 10 min at 37°C. Cell lysates were used for immunoprecipitation or immunoblot analysis with the indicated antibodies. (B) FN-mediated cell adhesion increases phosphorylation of EGFR, Akt and Erk. Cos7 cells were incubated in suspension (Sus) for 1 h and plated on FN for 30 min or 3 h. They were then lysed, subjected to SDS-PAGE and immunoblotted using the indicated antibodies. In all of our experiments, whole cell lysate was also imunoblotted as indicated, and tubulin was used as a loading control. (C) Overexpression of p130Cas enhances FN-induced phosphorylation of EGFR and Akt, but not Erk. Cos7 cells were transfected with empty vector (GFP-EV) or GFP-p130Cas (GFP-Cas) and treated as described in (B). (TIF) [file pone.0020125.s001.tif]
